# Illinois State Medical Society—A Contribution to the Report of the Committee on Medical Legislation

**Published:** 1882-06

**Authors:** E. Ingals

**Affiliations:** Chicago


### ARTICLE IV.

### A CONTRIBUTION TO THE REPORT OF THE COMMITTEE ON Medical LEGISLATION. (Read before the Illinois State Medical Society at Quincy, May, 1882). By E. INGALS M. D. Chicago.

That the paramount interests of civilized life require the fostering and protecting care of law, has been fully established by the aggregate experience of mankind, and certainly few things can be of greater importance to the human race than those that conserve its sanitary interests; and this, whether the subject is viewed in the light of the individual and family, or of the public good. A large percentage of the sickness that is suffered—and this not alone by the ignorant and indigent, but by the wealthy and intelligent as well—is preventable. Though centers of infection are apt to be first developed amid squalid surroundings, diseases thus engendered often escape from their place of origin to invade precincts that would otherwise have remained exempt. Ignorance of sanitary laws, or inattention to their observance is, however, by no means limited to the poorer classes.

So far as is possible, protection against all deleterious agencies, is a right, to which every citizen is entitled from organized society, and government should make the poisoning of the essentials of life and health a penal offense, and it should insure that those who are commissioned, under its laws, to the practice of medicine, should be reasonably well qualified for the proper discharge of the duties of their office. Our commerce, manufactures, agriculture, and common schools, are all regulated to some extent, by laws enacted in the supposed interests of the people.

We have also made some effort to promote the san-itary good of society, in the same manner, for in 1877 the legislature of Illinois passed a law to regulate the practice of medicine, and another to establish a State Board of Health. Hopes were entertained that great good would come from these enactments; especially as it was believed that their influence would increase the efficiency of the medical profession. These laws have now been in operation long enough to test their capacity to accomplish the purposes for which they were placed among our statutes, and our hopes have hardly been realized.

From a careful observation of the effects of these laws ever since they have been in force, I cannot see wherein they have done more than to secure a somewhat imperfect registration of the doctors and midwives of the State, and satisfactory mortuary returns. In spite of them, quacks practice with impunity, the same as before; revoking the license of a well known practitioner, who under the convenient disguise of an alias is guilty of the most unprofessional acts, does not prevent him from continuing his vocation; infectious diseases are not generally reported, and the returns of births are so incomplete that we might better make no pretense of collecting any such statistics. Our sanitary laws are not sufficiently explicit and comprehensive; they are not stringent enough; in some particulars they are obscure and they do not contain proper provisions for their enforcement. Increased security against the dangers of small pox should be insured by providing for a more complete system of vaccination; by enacting severe penalties for secreting such cases, and by prohibiting or regulating their transportation from place to place. The placing of warning cards to indicate the location of diseases that are infectious and highly dangereous, should be made compulsory, and public funerals in such cases should be prohibited by specific enactment.

Infected premises should be inspected by an official appointed for the purpose, who being clothed with authority, should be required to cause the removal of all insanitary conditions incident to the construction and surroundings of the place. These are only a few hints and suggestions of what might be embraced in the scope of the law.

Some legal means should also be taken to improve the character and increase the usefulness, of the medical profession. On this subject I wish not to be misunderstood. No one is more conscious than I am that the excellence of the medical profession, as represented by its most intelligent members, was never before as high as it is now, and it is every day improving. But while this is true, the facts we meet when we consider the other extreme of the profession are such as should stimulate us to an earnest effort for their removal. Two bodies cannot occupy the same place at the same time; and if the law should rule out incompetent applicants from the profession, the places from which they are excluded would be filled by those who are fitted to occupy them; but if the incompetent are once admitted, they can only be deprived of business by the competition of those who are more capable and better instructed, and the decision between them must be made by the tribunal of the general public, which does not possess qualifications properly to determine such a question.

I believe the standing and worth of the profession would be advanced, by the establishment of a State Board of Examiners, before which all who desire to practice in the State, and who should not already have been registered should go, for examination, and only to be allowed to practice after they had received a certificate of competency from such board. This board should be prohibited from granting license to practice to all who are not graduates from some medical college in good standing, and this good standing of any medical college should be determined by the legal requirement that it should exact of students certain literary and scientific qualifications, to be prescribed by the law, and four years study of medicine, and not less than three annual courses of college instruction, of not less than six months each, for graduation. This would not prejudice the interests of our medical colleges, but would place them all on the same basis, and lead to a generous and laudable rivalry between them as to which should give the best instruction, and this question would be determined by an impartial tribunal. Were such a plan adopted it would impose no hardship on our medical teachers, for they would receive the same rate for teaching as they now do, and if they were required to give more instruction they would be paid proportionately more for it. But should this not be the case, it would be no valid argument against the proposition; for so trifling a matter as the pecuniary interests of a few medical teachers should not be permitted to dominate the best interests of the whole people.

If the medical diploma is to continue to be allowed to confer on its possessor the right to practice medicine in Illinois, great discrimination should be exercised in bestowing it. At present the facility with which it may be obtained diminishes its value.

Though our State has made a great advance in numbers and wealth, our students have abundant means, and leisure for prolonged study, we continue in our requirements for ingress to the profession to follow the practices of a primitive state of society and suitable only for it. The time and money necessarily expended in gaining admission to the profession under the plan proposed would be increased but the physician's rewards would be greater after the admission was gained. Fifteen years of practice after thorough preparation is better for the physician than twenty years with an indifferent preparation, while the gain to society is much greater than to the physician.

Should Illinois do this, the schools of other States would be obliged to follow our advance, or we should educate all of our own practitioners, and while our graduates would occupy the entire field in Illinois, they would be preferred in other States, because they had received their medical education under a more thorough system of instruction.

It is well known that graduates from our medical schools who go before the army and naval boards for admission to the public service usually make special preparation for the examination, and yet, above eighty per cent. of such applicants are now being rejected. Surely no one will claim that citizens merit less skillful medical attendance than the soldiers and sailors of our army and navy. The excellent qualifications of recent graduates who present themselves for examination before our hospital boards for appointment as internes in these institutions is a subject of common remark. These places are coveted, and it is known that superior attainments are necessary in order to secure them, and the class of students who may indulge some hope of gaining them, work for them from the time they commence their medical studies. If there were fifty such places where there is now but one, there would still be no lack of well qualified graduates to fill them.

Raising the requirements of practitioners of medicine in Illinois would result in no void in the profession. It would only compel a certain number of persons to follow useful vocations outside of the profession, whose labors in the profession can be spared with mutual benefit to themselves and the public.

An easy and entirely practicable auxiliary for improving the character of the profession in Illinois, would be the establishment in some large city of the State, of a medical and dental department of the Illinois Industrial University which is located at Urbana. Illinois has been generous—as she should continue to be—with the charities. She provides liberally for the insane, the blind, the deaf, the feeble minded, and the convicts in her penal institutions. Would it not be just, wise, and politic, to exercise equal liberality towards the class of normal healthy minds that by higher cultivation would gain added powers of usefulness for the public good? The University at Ann Arbor, so far from having been a burden to Michigan, has doubtless been the means of increasing her wealth and numbers to a degree that would have made its founding and support an act of wisdom, if viewed only in the light of the State's material interest. If Michigan had never established this University her rank would be less advanced than it is at present among her sister States. But it is unfortunate that the medical department of the University had not been located at Detroit, for all medical schools, for obvious reasons, should be in large cities.

Illinois will have six or eight millions of people, who will possess an amount of wealth that we at present little realize, and we have greater cause for solicitude about our future moral and intellectual condition than for what pertains to our material interests. We should provide for a University that would in time take rank with any in the world. Such an institution would be of incalculable benefit, and would stamp its impress on our entire people.

The world is familiar with Dartmouth, Cambridge, New Haven, and Ann Arbor in America, with Oxford, Cambridge, Heidelburg, Göttingen, and many other cities of the old world; chiefly because they have been the seats of learning of superior excellence. If the Illinois Industrial University had a medical department, through this it might fix the standard of medical education for the State, for all the schools would necessarily advance their requirements until they would be recognized under the statutes by the board of examiners; because no school would be willing to issue diplomas that would not entitle their possessors to such an examination. The expense of such a department to the State need be nothing beyond the original plant, as fees from students, no higher than are now charged by other schools, would pay teachers of the first rank, meet all incidental expenses, and furnish it munificently with every means of teaching and illustration; and should it acquire the public confidence and sympathy by establishing and maintaining a high grade of excellence it would doubtless become the recipient of generous private endowments, as the medical departments of Harvard, and of the University of Pennsylvania have from a like cause.

A single appropriation by the State of one hundred thousand dollars, to be expended in building and equipping the institution, would be all that would be required to establish and maintain a medical department of our University that should be a credit to any State, for the school once in operation would provide abundantly for its every want from the fees of students. We annually graduate a larger number of medical students than we ought. The adoption of the recommendations we have offered would tend to decrease the number and improve the quality of those receiving the degree.

---

MALARIA IN RHODE ISLAND.—This State had been free from malaria for the past fifty years, until the summer of 1880. At this time an endemic of intermittent fever occurred at Nyatt. During the past summer, Dr. C. V. Chapin tried to estimate the extent of the disease in the city of Providence. He obtained reports of about 300 cases.—MED. REC.

